# Repetitive posterior iliac crest autograft harvest resulting in an unstable pelvic fracture and infected non-union: case report and review of the literature

**DOI:** 10.1186/1754-9493-1-6

**Published:** 2007-12-17

**Authors:** Matthew J Oakley, Wade R Smith, Steven J Morgan, Navid M Ziran, Bruce H Ziran

**Affiliations:** 1 Department of Orthopedic Surgery, Denver Health Medical Center, University of Colorado School of Medicine, 777 Bannock Street, Denver, CO 80204, USA; 2 Department of Orthopedic Trauma, Northeast Ohio Universities College of Medicine, St. Elizabeth Health Center, 1044 Belmont Ave, Youngstown, OH 44501, USA

## Abstract

Fractures of the pelvic ring have been well studied, and the biomechanical relationship between the anterior and posterior elements is an important concept to understand these complex injuries. The vast majority of these injuries are due to trauma. However, in rare circumstances, autogenous bone graft harvesting may lead to an unstable pelvic ring. In this case report, we describe a rare complication in a 70-year old female patient who developed an unstable pelvis and an infected non-union secondary to repeated posterior iliac graft harvest. The orthopaedic surgeon should be aware of this detrimental complication associated with extensive or repeated posterior iliac crest graft harvest.

## Introduction

The posterior iliac crest is frequently utilized for bone graft harvest to augment posterior spinal fusion surgery. Several authors have reported complications of autograft harvesting from this location. These range from minor complications reported in 10% to 39% of cases [1-6] to significant morbidity in 0% to 10% [1,2,7]. Common minor complications include iliac crest pain, hematoma, and sensory nerve palsy. Major complications included superior gluteal artery injury, sciatic nerve injury, deep wound infection, donor site infection and neuralgia parasthetica. Fractures of the iliac wing are known to occur, [1,8-10] but the majority are stable and surgical treatment has only been described in one case [11]. Pelvic instability secondary to removal of the posterior sacroiliac ligaments during the harvest of bone graft has been described and in some cases, operative intervention was required [12,13].

We describe the occurrence of an unstable iliac wing fracture extending into the sacroiliac joint, with associated disruption of the pubic symphysis complicated by infection and non-union following bone graft harvest of the posterior iliac crest. The potential of an iatrogenic iliac crest fracture to result in an infected non-union with a biomechanically unstable pelvic ring has not been described.

## Case presentation

**Figure 1:**
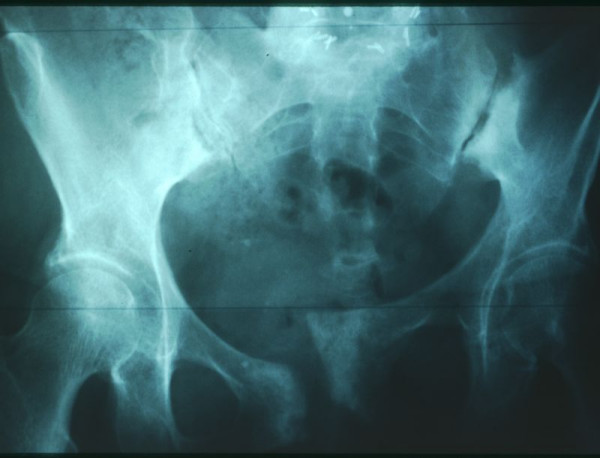
Antero-posterior X-ray of the pelvis demonstrating an unstable pelvic ring with anterior symphyseal disruption and rotation of the left hemipelvis due to an infected non-union secondary to an iatrogenic fracture after repeated posterior iliac crest bone graft harvesting.

**Figure 2:**
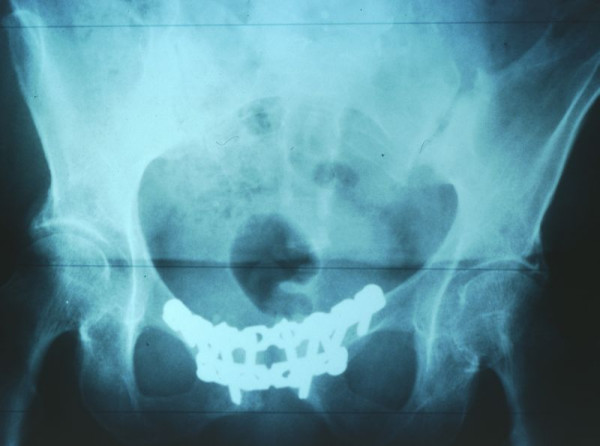
Postoperative antero-posterior X-ray of the pelvis demonstrating double-plating of the pubis symphysis with two 4.5 mm reconstruction plates.

A 70 year old woman with a history of low back pain and discogenic symptoms underwent open discectomy. Over the next year her back pain worsened, and her spine surgeon performed an L4-L5 *in situ *spinal fusion with posterior iliac crest autograft. She continued to experience progressive and disabling pain and subsequently underwent an L4-S1 fusion via both anterior and posterior approaches. During surgery an injury occurred to her inferior vena cava requiring extensive resuscitation and a sixty day stay in the intensive care unit. Post-operatively, a wound dehiscence and *Staphylococcus Aureus *infection developed in the posterior incision. Treatment consisted of wound debridement, dressing changes and intravenous antibiotics until wound healing. She subsequently recovered from surgery with low grade back pain but increasing pain in her gluteal region and symphysis pubis region. Six months after her fusion an AP x-ray view of the pelvis was obtained revealing symphysis diastasis with significant rotation of the left hemipelvis (Fig. 1). She was referred for treatment of the pelvic ring disruption. Reconstruction of the pelvic ring disruption was approached with double plating of her symphysis pubis with 4.5 mm reconstruction plates (Fig. 2). No surgical treatment of the posterior pelvis was undertaken. Two months following ORIF of the pubic symphysis she presented at a community hospital with anterior pelvic pain, wound drainage and fever. Aspiration of the suprapubic region revealed the presence of purulent material. She was transferred to the regional trauma center for further care. At the time of referral she was hemodynamically stable. Pain to palpation in her anterior pubis region and over her left sacroiliac joint was noted. Her white blood cell count was 11.2 × 10^9^/L, and her erythrocyte sedimentation rate was 52 mm/hr. A CT scan and radiographs revealed significant fluid collection in the area the symphysis and a complete dissociation of the left hemipelvis with erosion of the left iliac wing near the site of the posterior crest graft. Indium bone scan was unremarkable with no evidence of active uptake in the posterior pelvic ring.

**Figure 3:**
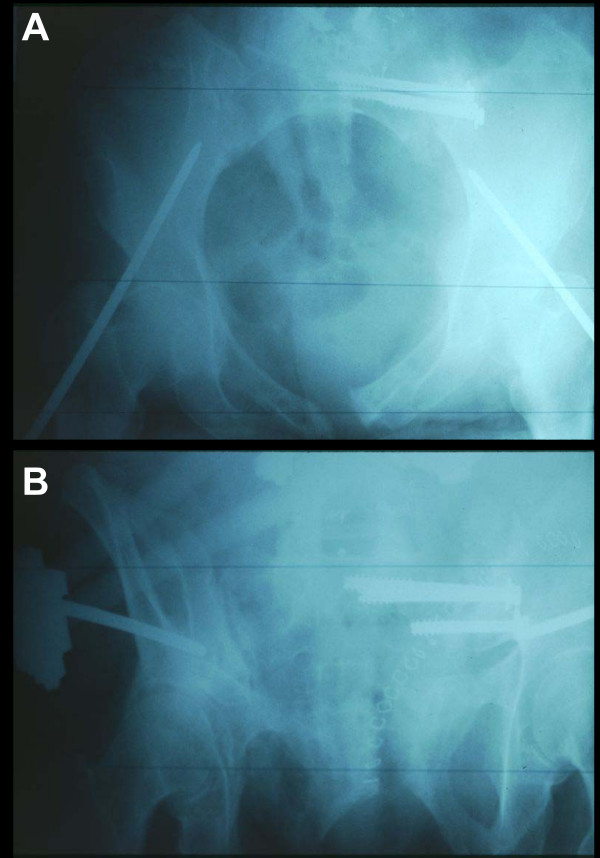
Pelvic inlet (panel **A**) and outlet (panel **B**) X-ray views after posterior sacro-iliac screw fixation and placement of an anterior external fixator for rotational control.

Initially, the anterior pelvis was debrided through her previous Pfannensteil incision. Forty milliliters of purulent fluid was evacuated and the plates were removed. Cultures were positive for coagulase-negative *Staphylococcus*. Based on culture and sensitivity studies intravenous vancomycin therapy was initiated and continued for 3 weeks. The posterior graft site was re-explored, and we found a large posterior iliac crest fracture. There was intra-operative evidence of both a malunion and non-union but no evidence of gross infection. Intraoperative gram stain was negative. The fractured segment was severely osteopenic, could not contribute to the stabilization, and was excised. The sacro-iliac joint was reduced, decorticated and grafted with allograft and demineralized bone matrix. The joint was reduced and compressed with three iliosacral screws between the sacrum and the remaining portion of the posterior ilium. The posterior fixation was supplemented by an anterior external fixator in order to control rotation (Fig. 3). Postoperative cultures revealed *Staphylococcus Aureus *despite the negative intra-operative gram stain. This bacteria was different than the bacteria found in the anterior pelvis but the same organism responsible for the previous spine infection. In view of the positive culture, the intravenous vancomycin was continued for three more weeks prior to conversion to oral rifampin and levofloxacin for a six month treatment course.

**Figure 4:**
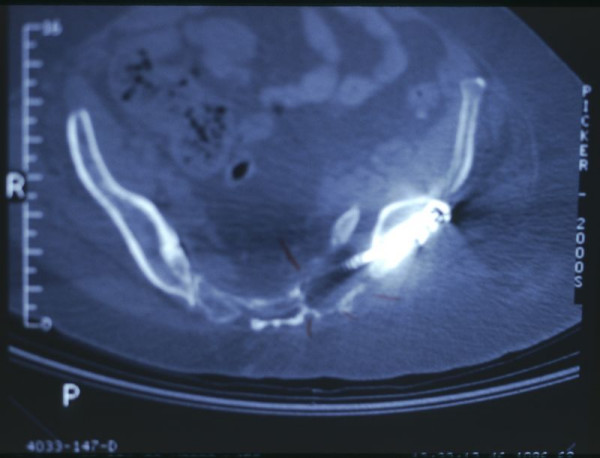
Axial CT scan of the posterior pelvic ring demonstrating the sacro-iliac screw in a deficient bone stock.

Post-operatively the patient continued to experience posterior pelvic pain and loosening of the iliosacral screws occurred by four weeks. The iliosacral screws were removed and repeat debridement of the non-union site with revision fixation was performed. Revision fixation was performed with the aid of CT guidance secondary to the lack of normal landmarks caused by bone loss (Fig. 4). These screws remained *in situ *for nine months before they also required to be removed due to loosening.

Currently, at two year follow up, the patient has minimal pain and is ambulatory with any aids or assistance. Her laboratory data indicates no signs of active infection and are within normal limits. Her radiographs demonstrate a stable fibrous union of the sacro-iliac and pubic symphyseal joints.

## Discussion

Autologous bone graft remains the gold standard in many orthopaedic conditions. The posterior iliac crest has been shown in some series to provide substantial quantities of bone with minimal morbidity and no fractures [2]. However, some series of autogenous bone graft harvest have reported substantial problems, such as fracture, nerve injury, and infection [1,7,9,14]. It has therefore been suggested that surgical technique plays a part. Improper location of the graft site and propagation of fracture lines from osteotomes are potential problems over which the surgeon has control. Ebraheim et al [15] showed that in a group of 24 patients with persistent donor site pain, 16 had CT evidence of injury of the sacroiliac joint. While the effect of osteotomes on the donor site has never been documented, they have been shown to significantly reduce the strength of the graft [16], and it is possible that similar mechanisms could occur to the remaining bone at the donor site.

Minor fractures have usually been described in women, and in the vast majority of these cases, these minor fractures could be treated without surgery [1,8-10]. Hu & Bohlman [9] suggested that fracture usually occurred in osteoporotic females. One case in the literature is described where the morphology of the fractures induced an unstable ring requiring operative intervention [5].

In the present case, repetitive graft harvest occurred at the same site in an elderly, osteoporotic female putting her at high risk for graft site complications. The posterior crescent fracture became an infected non-union, which likely led to a pubic symphysis disruption. This chain of events has been demonstrated before. Coventry et al and Lichtblau et al postulated that primary sacroiliac instability could lead to either stress fracture of the pubis or symphyseal diastasis [3,12]. These studies together with the case report by Fernando et al [11] suggest that the persistence of low back pain post graft harvest, with the onset of anterior pelvic pain, should warrant careful clinical examination and an x-ray series to exclude pelvic fracture. This advice could have speeded up the diagnosis in our case. The delayed diagnosis in the presence of infection caused changes in bone quality resulting in a technically difficult situation for definitive reconstruction.

The next point to be raised concerns the first attempt at treatment. Almost certainly the infected non-union at the back of the ring led to the symphysis diastasis [12,13], and therefore stabilizing the symphysis alone without addressing the posterior non-union was unlikely to succeed.

In summary, this case illustrates a devastating complication of repetitive bone graft harvest and the subsequent prolonged clinical course. Overall, there is evidence that with the appropriate clinical awareness and appropriate treatment care could have been significantly improved. Additionally, surgeons need to be aware of the potential for pelvic fracture as a complication of bone graft harvest, particularly in osteoporotic patients or after repetitive grafting. While autologous bone grafting remains the gold standard, it can have significant morbidity [1-3,5-12,14,15]. In the osteoporotic patient, repetitive harvest from the same site should be avoided or an alternative graft, such as anterior iliac crest, allogenic bone, or bone morphogenic protein-2, should be considered.

## Abbreviations

AP: Anterior-posterior;

CT: Computed tomography;

ORIF: Open reduction and internal fixation.

## Competing interests

The author(s) declare that they have no competing interests.

## Authors' contributions

MJO wrote the first draft of the manuscript based on a patient in his clinical practice. WRS, SJM, NMZ, and BHZ all contributed in revising the manuscript critically for scientific and clinical content and gave final approval.
